# Further records of social parasitic ants in Europe and review of the Bulgarian species

**DOI:** 10.3897/BDJ.12.e123575

**Published:** 2024-05-27

**Authors:** Albena Lapeva-Gjonova, Sándor Csősz, David Mifsud

**Affiliations:** 1 Department of Zoology and Anthropology, Faculty of Biology, Sofia University, 8 Dragan Tsankov str., Sofia, Bulgaria Department of Zoology and Anthropology, Faculty of Biology, Sofia University, 8 Dragan Tsankov str. Sofia Bulgaria; 2 HUN-REN-ELTE-MTM Integrative Ecology Research Group, Pázmány Péter ave 1/C, Budapest 1117, Hungary HUN-REN-ELTE-MTM Integrative Ecology Research Group, Pázmány Péter ave 1/C Budapest 1117 Hungary; 3 Department of Systematic Zoology and Ecology, Institute of Biology, ELTE-Eötvös Loránd University, Pázmány Péter ave 1/C, Budapest 1117, Hungary Department of Systematic Zoology and Ecology, Institute of Biology, ELTE-Eötvös Loránd University, Pázmány Péter ave 1/C Budapest 1117 Hungary; 4 Institute of Earth Systems, Division of Rural Sciences and Food Systems, University of Malta, Msida MSD 2080, Malta Institute of Earth Systems, Division of Rural Sciences and Food Systems, University of Malta Msida MSD 2080 Malta

**Keywords:** ants, inquilines, slave-makers, new records, fauna, conservation

## Abstract

**Background:**

Social parasitic ants exploit the colonies of other ant species, either permanently or temporarily. The permanent parasites are amongst the rarest species of ants, although their hosts may be very common. Due to their rarity and often restricted distribution range, most of them are listed as vulnerable. Filling in the gaps in geographical and host ranges will advance our understanding of the social parasitic lifestyle's origin and evolution.

**New information:**

This study reports the first records of the slave-makers *Myrmoxenusalgerianus* (Cagniant, 1968) for Europe (Italy, Sicily) and *M.ravouxi* (André 1896) for Albania and the inquiline *Anergatesatratulus* (Schenck, 1852) for Malta (Gozo). We also report new localities of *Camponotusuniversitatis* Forel, 1890 for Albania and *Myrmoxenusgordiagini* Ruzsky, 1902, *M.kraussei* (Emery, 1915) and *Anergatesatratulus* for Bulgaria. Diversity, type of parasite-host relationships, host range, distribution and conservation of social parasitic ant species in Bulgaria are discussed. Although social parasitic ants are still understudied in Bulgaria, they represent 21% of the regional ant fauna.

## Introduction

Amongst ants, several socially parasitic species depend on another ants (hosts) for survival and reproduction for all or part of their life cycle (Hölldobler and Wilson 1990, Buschinger 2009, Rabeling 2020). This parasitic lifestyle is used by at least 400 species (2.8%) from the 14,190 ant species currently known worldwide (Rabeling 2020, Bolton 2024). Most of the social parasitic species are found in temperate regions, where they represent up to 30% of ant diversity (Kutter 1968, Seifert 2018) and even up to 40% in Scandinavian and Baltic countries (Gray and Rabeling 2023 in Appendix S3). It is believed that, globally, their number is far higher, based on the high degree of endemism amongst them and data from Europe, where they are better explored and comprise a significant share of all ant species.

Obligate host-parasite interactions are commonly classified into four types according to the dependence of the parasite on the host for brood care and colony foundation: xenobiosis, temporary parasitism, dulosis or 'slavery' and the most advanced - inquilinism (Buschinger 2009, Seifert 2018). The species from the latter two types, being permanent parasites, are amongst the rarest species of ants (Heinze et al. 1993, Trontti et al. 2006), although their hosts can be very common. This is particularly true for highly specialised inquilines that rely on one or a few host species. Trontti et al. (2006) suggest that their rarity may be due to reduced dispersal ability and the host's resistance to them.

Hölldobler and Wilson (1990) point out that permanent parasitic ant species (especially the inquilines) in Europe are mainly found in mountainous or arid regions. Although Bulgaria is a small country (110,910 km^2^), it is home to the highest point on the Balkan Peninsula, Musala (2,925 m) and mountainous areas (above 600 m a.s.l.) occupy 30% of its territory (Penin 2012). The country’s climate is characterised by the mixed influences of a mild Mediterranean climate from the south and a continental climate from the north, resulting in cold and wet winters and dry and hot summers. In Bulgaria, even though the ant fauna is considered relatively well studied, with 197 species currently known (Lapeva-Gjonova and Antonova 2022, Csősz et al. 2023, Seifert et al. 2024), there are few reports of permanent social parasitic ants and the discovery of new ones and data on their distribution are still ongoing.

Filling the gaps in the geographical and host ranges of social parasitic species will further our understanding of the origin and evolution of social parasitism. This will also support efforts towards their protection as many of them are listed as vulnerable (Buschinger 1987, Mabelis 2007, IUCN 2024) falling into the following groups: 1) species with significantly isolated populations; 2) stenotopic species, mostly associated with xerothermic grassland or forest habitats; 3) boreomontane species; and 4) rare species with insufficient data on population size.

This study presents the first records of *Myrmoxenusalgerianus* (Cagniant, 1968) for Europe (Italy, Sicily), *M.ravouxi* (André, 1896) for Albania and *Anergatesatratulus* (Schenck, 1852) for Malta (Gozo Island). New localities of *Camponotusuniversitatis* Forel, 1890, *Myrmoxenusgordiagini* Ruzsky, 1902, *M.kraussei* (Emery, 1915) and *Anergatesatratulus* are also reported and the knowledge on diversity, type of parasite-host relationships, host range, distribution and conservation of 42 social parasitic ant species in Bulgaria is summarised.

## Materials and methods

The new data on the distribution of the species have been obtained as a result of several field entomological studies on the Maltese islands, Sicily (Italy), Albania and Bulgaria, using hand collection and pitfall traps. Unless otherwise stated, the material was collected by the first author and deposited in the Zoological Collection of the University of Sofia (BFUS). The first two authors carried out the identification of the ants using the latest identification keys, original species descriptions (Cagniant 1968, Kutter 1977, Cagniant and Espadaler 1997, Czechowski et al. 2012, Seifert 2018) and type material on AntWeb (2024). Images of the specimens were taken by AL-G with Nikon DS-Ri2 through Nikon SMZ 1270i stereomicroscope and then aligned and stacked using CombineZ free software.

## Taxon treatments

### Anergates atratulus

(Schenck, 1852)

FF0250A9-56FE-5EC4-A680-A90912002103

#### Materials

- **Type status:** Other material. **Occurrence:** catalogNumber: BFUS-I-AG001990; occurrenceRemarks: found together with the host workers, males and queens of *Tetramoriumsemilaeve* André, 1883; recordedBy: A. Lapeva-Gjonova; individualCount: 1; sex: female; reproductiveCondition: physiogastric; preparations: EtOH; occurrenceID: 6A8EC018-7368-5DA5-BD91-338261A9811D; **Taxon:** scientificName: *Anergatesatratulus* (Schenck, 1852); order: Hymenoptera; family: Formicidae; taxonRank: species; **Location:** island: Gozo; country: Malta; locality: Ramla Bay; minimumElevationInMeters: 15; locationRemarks: under stone; decimalLatitude: 36.0601; decimalLongitude: 14.2834; **Event:** eventDate: 06-06-2019; habitat: littoral; **Record Level:** collectionID: BFUS; basisOfRecord: PreservedSpecimen
- **Type status:** Other material. **Occurrence:** catalogNumber: BFUS-I-AG001992; occurrenceRemarks: found together with the host workers of *Tetramoriumimmigrans* Santschi, 1927; recordedBy: A. Lapeva-Gjonova; individualCount: 11; sex: 10 queens, 1 male; preparations: EtOH; occurrenceID: 88345A4A-2981-5BCC-B0BB-9C3DE3843FB5; **Taxon:** scientificName: *Anergatesatratulus* (Schenck, 1852); order: Hymenoptera; family: Formicidae; taxonRank: species; **Location:** country: Bulgaria; county: Burgas; municipality: Malko Tarnovo; locality: near Kalovo vill.; minimumElevationInMeters: 387; locationRemarks: under stone; decimalLatitude: 42.14638; decimalLongitude: 27.54572; **Event:** eventDate: 06-06-2021; habitat: along a road in an oak forest; **Record Level:** collectionID: BFUS; basisOfRecord: PreservedSpecimen

#### Notes

This is the first record for Malta and is likely the southernmost distribution point of this workless inquiline species in Europe. *Anergatesatratulus* has a native range that covers the western Palaearctic, where it uses several ant hosts, usually from the *Tetramoriumcaespitum* species complex, but also from the *T.ferox* and *T.chefketi* complexes (Sanetra et al. 1999, Lapeva-Gjonova et al. 2012, Wagner et al. 2017, Seifert 2018, Purkart et al. 2022). In Malta, the host of *Anergatesatratulus* appears to be *Tetramoriumsemilaeve*, which has recently been reported for the first time as a new host of this social parasite in Sardinia (Schifani et al. 2021b). This rare workless inquiline was found in a littoral zone of Gozo Island as a physiogastric queen and pupae under a stone together with workers and numerous males and alate queens of the ant host (Figs 1, 2). A similar unusual finding of *A.atratulus* together with workers and pupae of the host species (*T.diomedeum* Emery, 1908) was also reported by Sanetra et al. (1999) in Sicily (Siracusa distr.) at about 300 m a.s.l. in mid-May. As *Tetramoriumsemilaeve* is a typical western Mediterranean species (Borowiec et al. 2015), *A.atratulus* probably occurs naturally on the Island of Gozo. The late record of this species in the Maltese Islands is explained by its rarity, highly anthropogenised habitats and the increasing threat of invasive ant species.

In the same nest of *T.semilaeve* with *A.atratulus*, apterous flat morphs of *Paracletuscimiciformis* von Heyden, 1837 (Hemiptera, Aphididae) were found. It is an aphid with a complex cycle on *Pistacia* (primary host) and grass roots (secondary host) in ant nests, mostly of *Tetramorium*, where they also prey on ants, sucking out the haemolymph of the ant brood with their stylets (Salazar et al. 2015).

In Bulgaria, it was reported from several regions without precise collecting localities (Atanassov and Dlusskij 1992) – Western Stara Planina Mts, Vitosha Mt., Osogovo Mt., Rhodopes Mts, Black Sea coast as well as from two exact sites in Konyavska Mt. and Eastern Rhodopes (Lapeva-Gjonova et al. 2012). The current finding is from Strandzha Mt. (south-eastern Bulgaria) in the nest of *T.immigrans*, which is the first record of this host for *A.atratulus* in Bulgaria.

### Myrmoxenus algerianus

(Cagniant, 1968)

E800BF89-415D-5862-9890-333A211B41A8

#### Materials

- **Type status:** Other material. **Occurrence:** catalogNumber: BFUS-I-AG001991; recordedBy: A. Lapeva-Gjonova; individualCount: 1; sex: queen; preparations: pinned; occurrenceID: 3239BA51-C917-5DBC-8967-675F377E919A; **Taxon:** scientificName: *Myrmoxenusalgerianus* (Cagniant, 1968); order: Hymenoptera; family: Formicidae; taxonRank: species; **Location:** island: Sicily; country: Italy; locality: Cassaro; minimumElevationInMeters: 317; decimalLatitude: 37.10299; decimalLongitude: 14.96467; **Event:** samplingProtocol: leaf-litter sifting; eventDate: 06-04-2015; habitat: karst gorge; **Record Level:** collectionID: BFUS; basisOfRecord: PreservedSpecimen

#### Notes

This is the first record for Europe. *Myrmoxenusalgerianus* is known from the Atlas and Rif mountains in Algeria (type locality) and Morocco (Cagniant 1968). Buschinger et al. (1990) found it in both deciduous and coniferous (cedar) forests at 400-2100 m a.s.l. It is an active enslaver, most often on *Temnothoraxspinosus* (Forel, 1909), but also on *T.curtulus* (Santschi, 1929), *T.gentilis* (Santschi, 1923) and *T.monjauzei* (Cagniant, 1968) (Buschinger 1989). Up to now, *M.ravouxi* (André 1896) has been the only known *Myrmoxenus* species from Sicily (Schifani et al. 2021a, Schifani 2022). *Myrmoxenusalgerianus* differs from congeners in the shape of the ventral subpetiolar process (almost rectangular rather than almost triangular in *M.kraussei*), the pilosity of the thorax (shorter in *M.algerianus* than in *M.kraussei*) and the position and length of the propodeal spines compared to *M.ravouxi* (Cagniant and Espadaler 1997) (Fig. 3a). The collected specimen in Sicily was found in a karst gorge by leaf-litter sifting (Fig. 3b). Although we have no information on the host from the new locality, none of the known host species from Morocco and Algeria occurs in Sicily. The following *Temnothorax* species were identified from the new locality: *T.recedens* (Nylander, 1853), *T.poldii* Alicata, Schifani & Prebus, 2022 and *T.lichtensteini* (Bondroit, 1918).

### Myrmoxenus ravouxi

(André 1896)

AAE96BCC-355D-5AF0-8320-96A30CF007C5

#### Materials

- **Type status:** Other material. **Occurrence:** catalogNumber: BFUS-I-AG001993; recordedBy: A. Lapeva-Gjonova; individualCount: 1; sex: worker; preparations: pinned; occurrenceID: C4786BB7-43EE-570B-8B59-4C266D1D33C1; **Taxon:** scientificName: *Myrmoxenusravouxi* (André 1896); order: Hymenoptera; family: Formicidae; **Location:** country: Albania; county: Korce; locality: Ostrovicë Mt., Voskopoje; minimumElevationInMeters: 1312; decimalLatitude: 40.644715; decimalLongitude: 20.60356; **Event:** eventDate: 07-07-2022; habitat: pine forest; **Record Level:** collectionID: BFUS; basisOfRecord: PreservedSpecimen

#### Notes

This is the first record for Albania and the first member of *Myrmoxenus* for this country. It is widespread in Europe (missing in northern parts) to eastern Turkey (Schulz and Sanetra 2002). It is an active slave-maker, most often on *Temnothoraxunifasciatus* (Latreille, 1798) (Buschinger and Winter 1985, Schulz and Sanetra 2002), but depending on the local fauna, it can also be found in nests of *T.affinis* (Mayr, 1855), *T.albipennis* (Curtis, 1854), *T.tuberum* (Fabricius, 1775) and *T.nadigi* (Kutter, 1925) (Czechowski and Czechowska 2000, Seifert 2018). The new finding of *M.ravouxi* is from a nest of *T.unifasciatus* in a pine forest in the Ostrovicë Mountain (southern Albania).

### Myrmoxenus gordiagini

Ruzsky, 1902

6CBAB83E-43A7-56B6-BD06-9672E7506369

#### Materials

- **Type status:** Other material. **Occurrence:** catalogNumber: BFUS-I-AG001994; recordedBy: M. Langourov; individualCount: 1; sex: queen; preparations: pinned; occurrenceID: 2FEA08F5-DEFF-543A-8CD4-1A0D73257F30; **Taxon:** scientificName: *Myrmoxenusgordiagini* Ruzsky, 1902; order: Hymenoptera; family: Formicidae; taxonRank: species; **Location:** country: Bulgaria; county: Blagoevgrad; municipality: Strumyani; locality: Struma Valley, near Kamenitsa vill.; minimumElevationInMeters: 170; maximumElevationInMeters: 240; decimalLatitude: 41.65; decimalLongitude: 23.167; **Event:** samplingProtocol: tree trap; startDayOfYear: 27-09-2002; endDayOfYear: 02-11-2002; habitat: xerothermophilic site with *Quercuscoccifera* L.; **Record Level:** collectionID: BFUS; basisOfRecord: PreservedSpecimen

#### Notes

This is an active slave-making species on some *Temnothorax* species – *T.lichtensteini* (Bondroit, 1918), *T.graecus* (Forel, 1911) *T.korbi* (Emery, 1924), *T.bulgaricus* (Forel, 1892) and *T.serviculus* (Ruzsky, 1902) (Buschinger and Douwes 1993, Schulz and Sanetra 2002, Bračko 2010). There was only one previous record of *Myrmoxenusgordiagini* from Bulgaria (Western Predbalkan, Reselets vill.) (Buschinger and Douwes 1993).

### Myrmoxenus kraussei

(Emery, 1915)

AD2F92EF-7F62-52FD-B5F2-F8036D5555ED

#### Materials

- **Type status:** Other material. **Occurrence:** catalogNumber: BFUS-I-AG001995; recordedBy: M. Langourov; individualCount: 1; sex: queen; preparations: pinned; occurrenceID: D271AAA3-2B74-5B38-BC98-9D5F798A89A3; **Taxon:** scientificName: *Myrmoxenuskraussei* (Emery, 1915); order: Hymenoptera; family: Formicidae; taxonRank: species; **Location:** country: Bulgaria; county: Blagoevgrad; municipality: Strumyani; locality: Struma Valley, near Kamenitsa vill.; minimumElevationInMeters: 170; maximumElevationInMeters: 240; decimalLatitude: 41.650; decimalLongitude: 23.167; **Event:** samplingProtocol: soil traps; startDayOfYear: 07-09-2002; endDayOfYear: 27-09-2002; habitat: xerothermophilic site with *Quercuscoccifera* L.; **Record Level:** collectionID: BFUS; basisOfRecord: PreservedSpecimen
- **Type status:** Other material. **Occurrence:** catalogNumber: BFUS-I-AG001995; recordedBy: M. Langourov; individualCount: 1; sex: worker; preparations: pinned; occurrenceID: 39583CA7-6A53-55DC-8F37-7F1094C33064; **Taxon:** scientificName: *Myrmoxenuskraussei* (Emery, 1915); order: Hymenoptera; family: Formicidae; taxonRank: species; **Location:** country: Bulgaria; county: Blagoevgrad; municipality: Strumyani; locality: Struma Valley, near Kamenitsa vill.; minimumElevationInMeters: 170; maximumElevationInMeters: 240; decimalLatitude: 41.650; decimalLongitude: 23.167; **Event:** samplingProtocol: soil traps; startDayOfYear: 07-09-2002; endDayOfYear: 27-09-2002; habitat: xerothermophilic site with *Quercuscoccifera* L.; **Record Level:** collectionID: BFUS; basisOfRecord: PreservedSpecimen

#### Notes

Although the species was considered a degenerate slave-maker because of its very low worker number, Suefuji and Heinze (2015) suggest that it may be actively involved in slave raiding. *Myrmoxenuskraussei* has a Mediterranean distribution and prefers xerothermophilic habitats, where it most commonly uses *Temnothoraxrecedens* as a host (Buschinger 1989, Tinaut et al. 2005). Only two records of *M.kraussei* were recently reported from Bulgaria (Vrachanska Mt.) (Ljubomirov 2019). It is well distinguished by long, pointed setae (145-170 μm) on the body.

### Camponotus universitatis

Forel, 1890

32034CC2-408D-5C89-BD52-F96D37FBBBC0

#### Materials

- **Type status:** Other material. **Occurrence:** catalogNumber: BFUS-I-AG001023 (EtOH), BFUS-I-AG001996 (pinned); occurrenceRemarks: found with *Camponotusaethiops* (Latreille, 1798) under a stone; recordedBy: A. Lapeva-Gjonova; individualCount: 6; sex: workers; preparations: 6 in EtOH, 1 pinned; occurrenceID: 79702482-B056-5A3A-A669-4C2DA01699BE; **Taxon:** scientificName: *Camponotusuniversitatis* Forel, 1890; order: Hymenoptera; family: Formicidae; taxonRank: species; **Location:** country: Albania; county: Gjirokastër; municipality: Përmet; locality: Frasher 3; minimumElevationInMeters: 1365; decimalLatitude: 40.35624; decimalLongitude: 20.41197; **Event:** samplingProtocol: hand collecting; eventDate: 18-06-2023; habitat: pine forest; **Record Level:** basisOfRecord: PreservedSpecimen

#### Notes

The species is an inquiline in the nests of *Camponotusaethiops* (Latreille, 1798) and *C.pilicornis* Roger, 1859, with few known localities in southern Europe (Spain, France, Italy, Switzerland, Albania, Bulgaria) and Anatolia (Karaman et al. 2015). It is clearly distinguished from other European species of *Camponotus* by its small size (about 5 mm in workers) and shiny body with standing claviform setae, which are also present on the dorsal side of the legs (Fig. 4a). The new finding of *C.universitatis* comes from a nest of the typical ant host for the Balkans, *C.aethiops*, in a pine forest of the Hotova National Park (Gjirokastër County) (Fig. 4b). After Andoni (1977), who first reported the species from two localities in Albania (Vlorë and Nivicë), this is a new record for this country after almost 50 years.

## Discussion

### Social parasitic ant species in Bulgaria - taxon diversity

Currently, 42 out of the 197 ant species found in Bulgaria show traits of a parasitic lifestyle (Table 1). They belong to three of the six subfamilies in Bulgaria – Formicinae (25 species), Myrmicinae (15 species) and Dolichoderinae (2 species), although the species richness for the country is reversed in the first two subfamilies, with Formicinae having 73 species and Myrmicinae 107 species (Lapeva-Gjonova and Antonova 2022, Csősz et al. 2023). Out of the 43 ant genera in Bulgaria, 14 contain species with a social parasitic lifestyle. Most belong to *Lasius* (13) and *Formica* (9), which are almost all temporary parasites, followed by the dulotic ant species of *Strongylognathus* (6) and *Myrmoxenus* (3). *Bothriomyrmex* has two temporary parasitic species, while *Myrmica*, *Harpagoxenus*, *Formicoxenus*, *Chalepoxenus*, *Anergates*, *Teleutomyrmex*, *Plagiolepis*, *Camponotus* and *Polyergus* are each represented by one species.

### Type of parasite-host relationships

Parasite-host relationships, most often referred to as four types: xenobiosis, temporary parasitism, slavery and inquilism (the most advanced), are represented amongst Bulgarian ants by 1, 24, 13 and 4 species, respectively. The typical xenobiotic species (guest ant) in Europe, which lives in the colonies of the host (*Formica* s.str.), but rears its own offspring separately, is *Formicoxenusnitidulus*. This species is rarely recorded because of its small size and numerous members of host colonies. Slightly more than half (57%) of the species of socially parasitic ants in Bulgaria are temporary parasites in the nests of other ants, which is close to the 50% reported for global ant parasite diversity (Borowiec et al. 2021). In these species, the parasitic queen enters the host colony, kills the resident queen and exploits the host workers to rear her offspring until they die. Twenty-one out of the 24 temporary parasitic species are members of the Holarctic genera of *Formica* (subgenera *Formica* and *Coptoformica*) and *Lasius* (formerly subgenera *Austrolasius*, *Chthonolasius* and *Dendrolasius*) and two are *Bothriomyrmex* species (*B.corsicus*, *B.communistus*). Another species, *Myrmicavandeli*, is often considered a temporary parasitic ant, although it exhibits both parasitism in poor or new habitats and independent colony foundation in optimal environmental conditions (Radchenko and Elmes 2010).

The other two types of parasite-host relationships are permanent and involve a series of morphological (e.g, broad head with strong or sabre-like mandibles) and behavioural adaptations (e.g. stinging, throttling, scouting or organised raids, chemical mimicry, expelling propagic compounds) to a limited host range (D'Ettorre and Heinze 2001). So far, 13 ant species in Bulgaria are dulotic social parasites, meaning that the parasites invade the host colony, kill the queen, steal resident workers to work for them and continue to take slaves by raiding neighbouring colonies and stealing offspring. However, in two species, *Strongylognathustestaceus* and *S.karawajewi*, slave raiding has definitely degenerated and in *Formicasanguinea* dulosis is facultative.

There are only four species of the most specialised permanent parasites, the inquilines, in Bulgaria (*Anergatesatratulus*, *Teleutomyrmexbuschingeri*, *Plagiolepisxene*, *Camponotusuniversitatis*), most of them (the former three species) having lost the ability to produce their own workers and, thus, depend directly on the host workers to raise the next generations of reproductive individuals. While *Teleutomyrmexbuschingeri* and *Plagiolepisxene* are known to be host-queen tolerant and *Anergatesatratulus* typically invades queenless colonies, there are no confirmed data on the host-queen attitude of *Camponotusuniversitatis* (Seifert 2018).

### Geographical distribution

Biogeography of social parasites depends on the distribution of their hosts and such data contribute to understanding the evolution of parasitic life histories. It was found that the proportion of ant social parasite species in the regional fauna is closely related to the latitude only in the Northern Hemisphere (Gray and Rabeling 2023).

Social parasitic ants in Bulgaria represent 21% of all species known for the country (197), as it is expected that already described species with large gaps in their distribution might also be found (e.g. *Leptothoraxkutteri* Buschinger, 1965, *L.goesswaldi* Kutter, 1967, *Myrmicahirsuta* Elmes, 1978, *Plagiolepisampeloni* (Faber, 1969)). This number is lower than the proportion observed in northern latitudes in Europe, but higher compared to some southern European countries, such as Greece and Spain, where ant diversity is much higher and well studied (Gray and Rabeling 2023).

It is not surprising that the temporary parasites recorded from Bulgaria (24 species) belong to only two major biogeographical complexes, mainly the Palaearctic (19 species - 9 Euro-West Siberian, 5 West Palaearctic and 5 Palaearctic), but also to the European (5 species). This is due to the representatives of the two genera – *Formica* (*Formica* s. str. and *Coptoformica* subgenera) and *Lasius*, which are associated with temperate to boreal-montane zones.

The biogeographical affiliation of the permanent parasites in Bulgaria (17 species) presents a different situation. In addition to the Palaearctic and European complexes, each with five species, three species have a Mediterranean distribution and four have a very limited range - one in Bulgaria only (*Teleutomyrmexbuschingeri*), two in the Balkans (*Strongylognathusbulgaricus*, *S.huberidalmaticus*) and one in the Ponto-Caspian Region (*S.karawajewi*).

### Conservation

Many social parasites are included in the IUCN Red List of Threatened Species (IUCN 2024) due to their dependence on host species and restricted range (Alonso 2009). In addition, they may be scarce, endemic or overlooked in field studies. Out of the 18 ant species in the Bulgarian myrmecofauna listed in the IUCN Red List of Threatened Species, 17 are social parasites. Eleven species of slave-makers and inquilines (permanent parasites) and *Lasiusreginae* (temporary parasite) are in the Vulnerable category. At the same time, five of the red wood ants are listed as Near Threatened or Least Concern.

There is currently no Red List of Bulgarian ants that reflects their conservation status and trends. The only national initiative with a conservation approach in Bulgaria that includes socially parasitic ant species refers to the developed monitoring and assessment methodologies for only two of the species - *Anergatesatratulus* and *Polyergusrufescens* (National System for Environmental Monitoring). An updated assessment of the conservation status of the regional myrmecofauna is needed to take into account both status and taxonomic changes (Antonova and Marinov 2021, Lapeva-Gjonova and Antonova 2022). Thus, potential candidates, such as *Strongylognathusbulgaricus*, *S.huberidalmaticus* and *Teleutomyrmexbuschingeri*, should be listed, as are their congeners. However, estimation of population sizes and ranges for these and many other invertebrates implies the introduction of potential new IUCN criteria to reflect more accurately their conservation needs (Goodsell et al. 2024).

## Supplementary Material

XML Treatment for Anergates atratulus

XML Treatment for Myrmoxenus algerianus

XML Treatment for Myrmoxenus ravouxi

XML Treatment for Myrmoxenus gordiagini

XML Treatment for Myrmoxenus kraussei

XML Treatment for Camponotus universitatis

## Figures and Tables

**Figure 1a.**
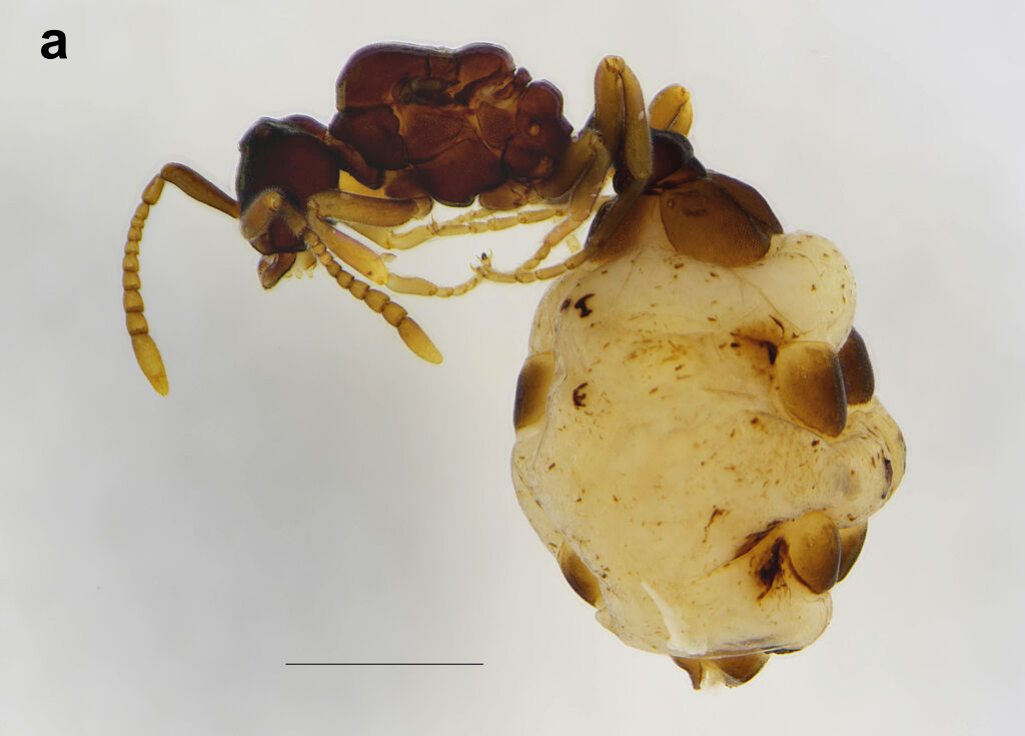
physiogastric queen, scale: 1 mm;

**Figure 1b.**
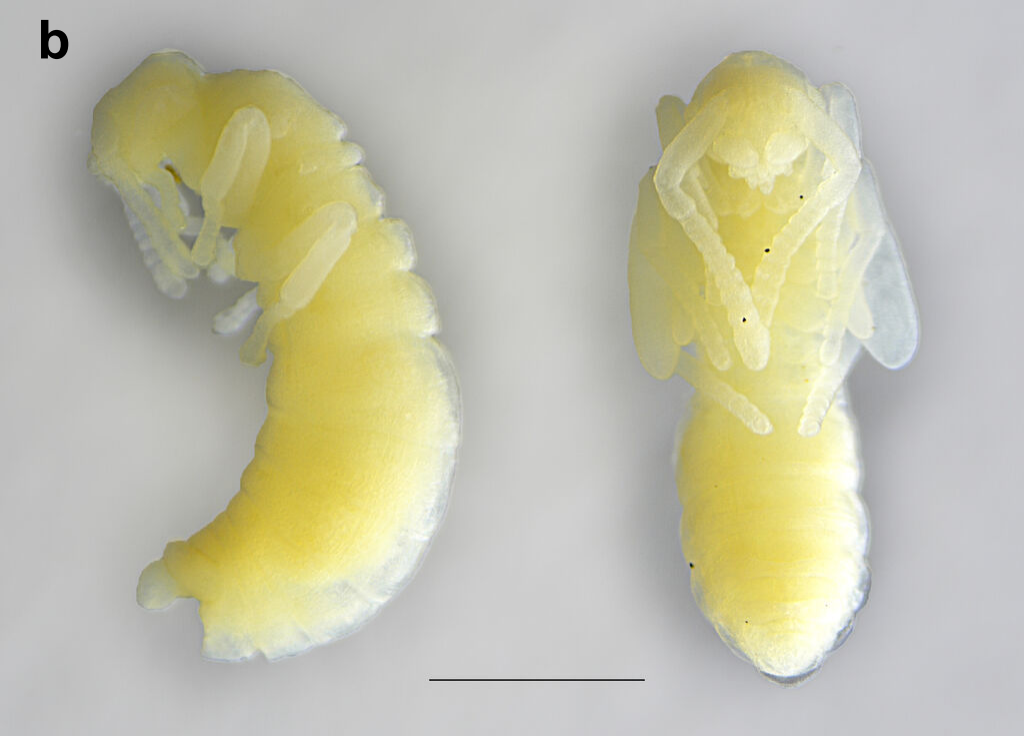
pupae - a male (left) and a queen (right), scale: 1 mm.

**Figure 2a.**
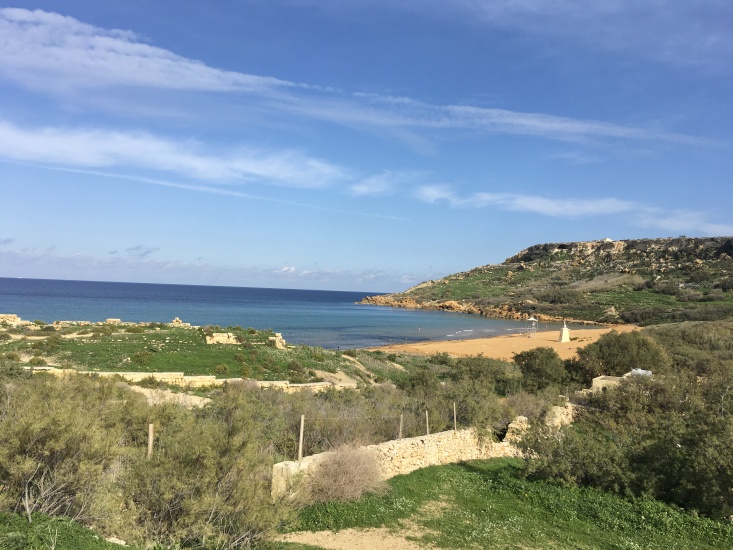
general view of the habitat;

**Figure 2b.**
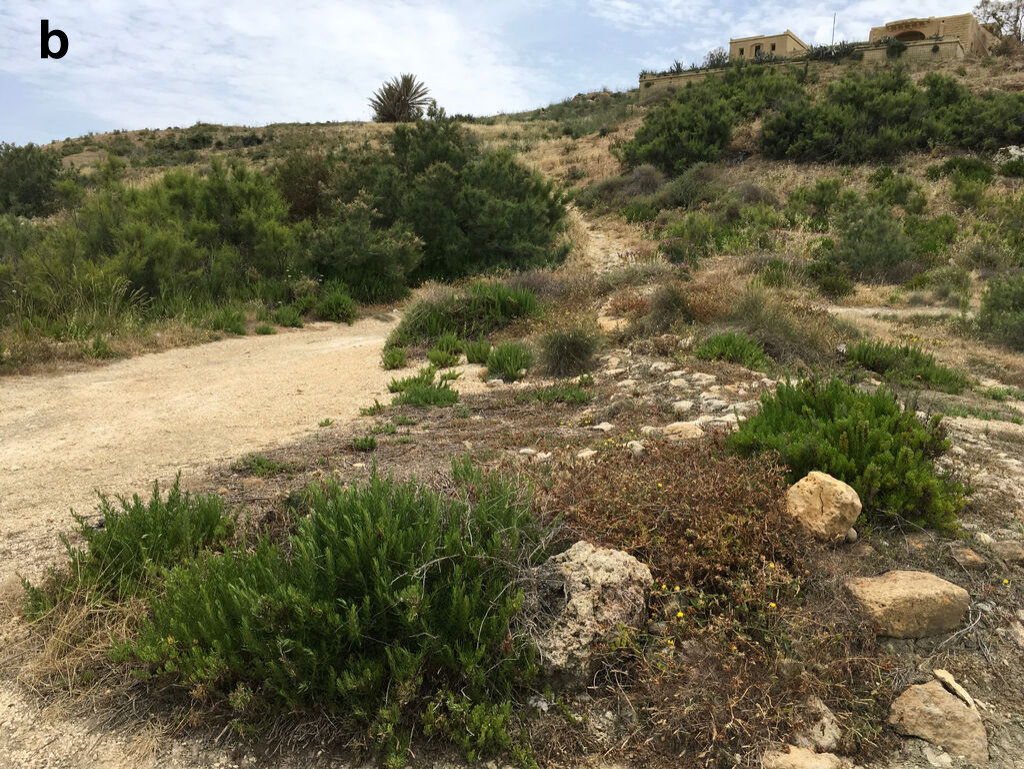
exact site of collecting.

**Figure 3a.**
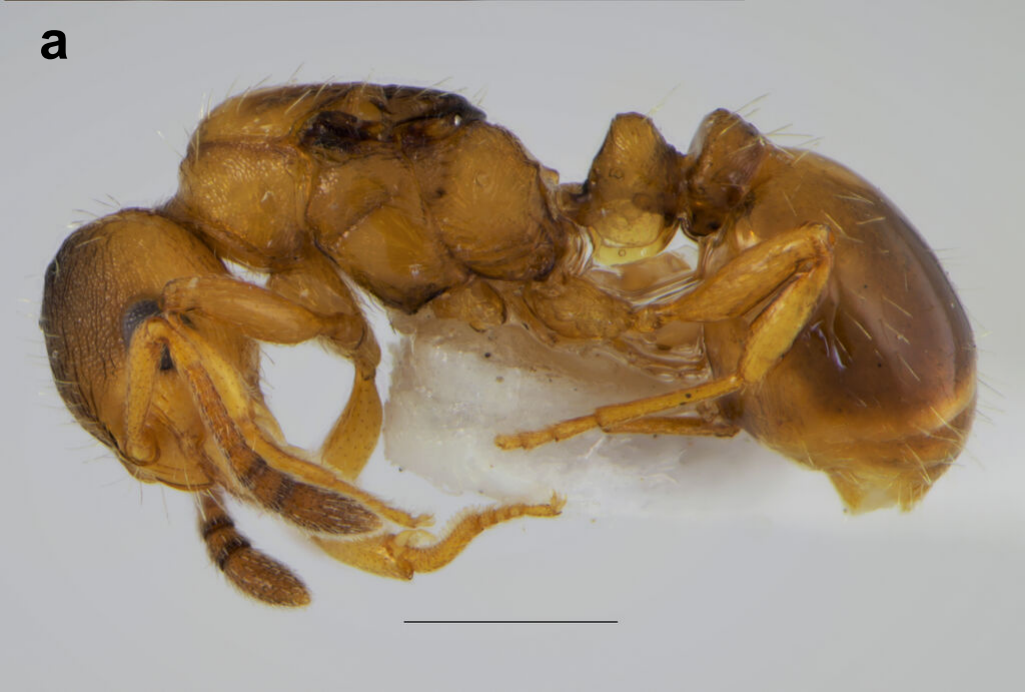
queen, scale: 1 mm;

**Figure 3b.**
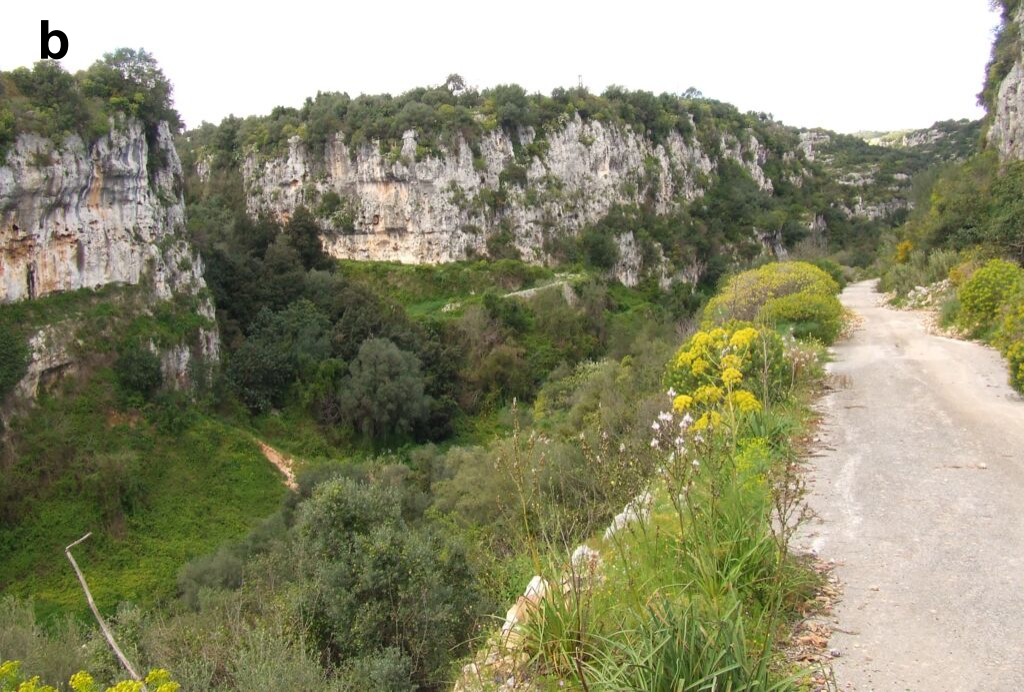
collecting site in Sicily; photo: R. Kostova.

**Figure 4a.**
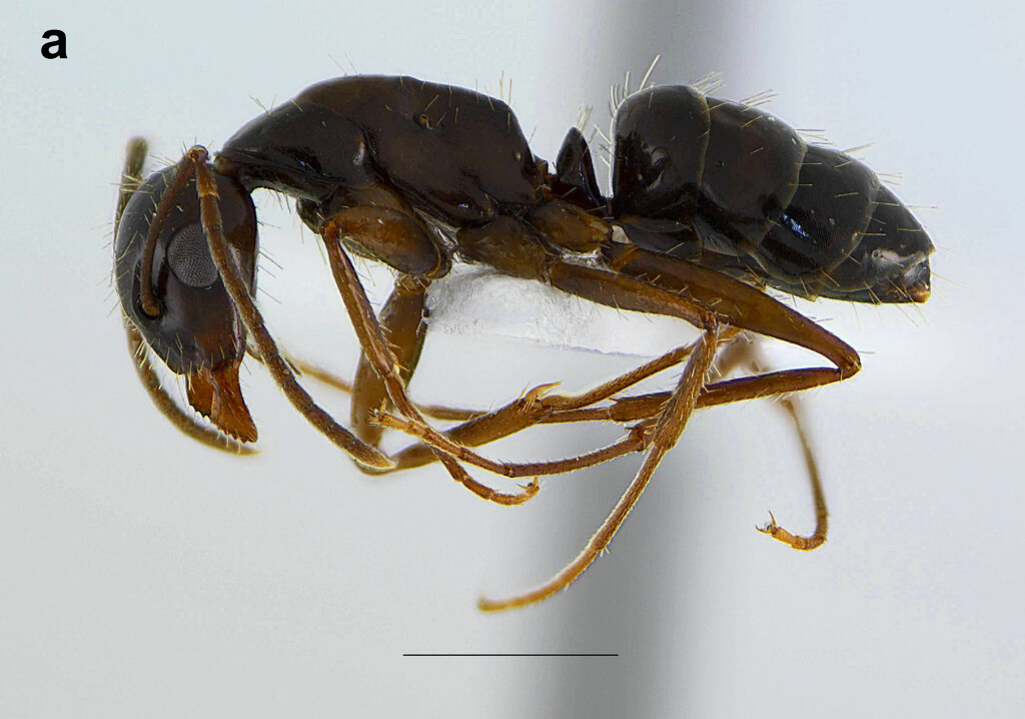
worker, scale: 1 mm;

**Figure 4b.**
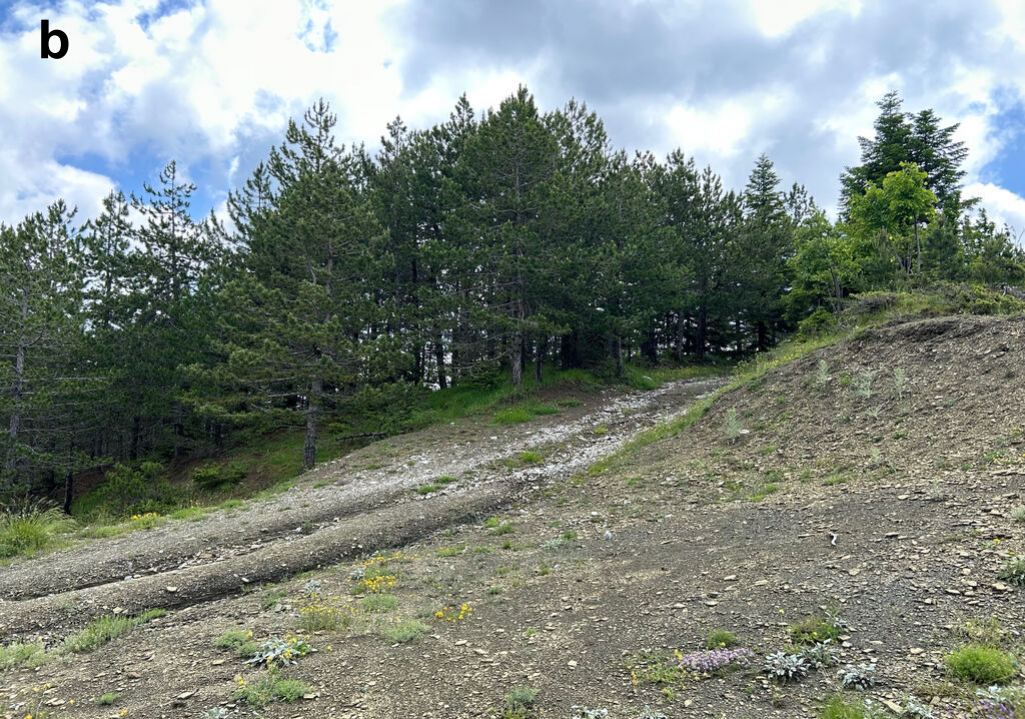
collecting site in Albania.

**Table 1.** Ant species with social parasitic traits known from Bulgaria listed by subfamily and tribe affiliation. For the host species, the ones reported from the country are given or, if such data are not available, the potential host species that occur in Bulgaria. “Vu” and “LR/NT” following the species name indicates the conservation status.

**Parasitic species / subfamily / tribe**	**Types of parasitism**	**Host species**
***Myrmicavandeli* Bondroit, 1920** Myrmicinae/Myrmicini	temporary	*Myrmicascabrinodis* Nylander, 1846 Myrmicinae/Myrmicini
***Harpagoxenussublaevis* (Nylander, 1849)** Vu Myrmicinae/Crematogastrini	dulosis	*Leptothoraxacervorum* (Fabricius, 1793) *Leptothoraxmuscorum* (Nylander, 1846) Myrmicinae/Crematogastrini
***Formicoxenusnitidulus* (Nylander, 1846)** Vu Myrmicinae/Crematogastrini	xenobiosis	*Formica* s.str. Formicinae/Formicini
***Myrmoxenusgordiagini* Ruzsky, 1902** Vu Myrmicinae/Crematogastrini	dulosis	*Temnothoraxlichtensteini* (Bondroit, 1918) Myrmicinae/Crematogastrini
***Myrmoxenuskraussei* (Emery, 1915)** Vu Myrmicinae/Crematogastrini	dulosis (degenerated?)	*Temnothoraxrecedens* (Nylander, 1856) Myrmicinae/Crematogastrini
***Myrmoxenusravouxi* (André, 1896)** Vu Myrmicinae/Crematogastrini	dulosis	*Temnothoraxaffinis* (Mayr, 1855) *Temnothoraxinterruptus* (Schenck, 1852) *Temnothoraxtuberum* (Fabricius, 1775) *Temnothoraxunifasciatus* (Latreille, 1798) Myrmicinae/Crematogastrini
***Chalepoxenusmuellerianus* (Finzi, 1922)** Vu Myrmicinae/Crematogastrini	dulosis	*Temnothoraxunifasciatus* (Latreille, 1798) Myrmicinae/Crematogastrini
***Anergatesatratulus* (Schenck, 1852)** Vu Myrmicinae/Crematogastrini	inquilinism	*Tetramorium* spp. Myrmicinae/Crematogastrini
***Teleutomyrmexbuschingeri* Lapeva-Gjonova, 2017** Myrmicinae/Crematogastrini	inquilinism	Tetramoriumcf.chefketi Myrmicinae/Crematogastrini
***Strongylognathustestaceus* (Schenck, 1852)** Myrmicinae/Crematogastrini	dulosis (degenerated)	*Tetramorium* spp. (*caespitum* complex) Myrmicinae/Crematogastrini
***Strongylognathuskarawajewi* Pisarski, 1966** Vu Myrmicinae/Crematogastrini	dulosis (degenerated)	*Tetramorium* sp. (*caespitum* complex) *Tetramoriumhungaricum* Röszler, 1935 *Tetramoriumchefketi* Forel, 1911 Myrmicinae/Crematogastrini
***Strongylognathusbulgaricus* Pisarski, 1966** Myrmicinae/Crematogastrini	dulosis	*Tetramorium* spp. (*caespitum* complex) Myrmicinae/Crematogastrini
***Strongylognathushuberidalmaticus* Baroni Urbani, 1969** Myrmicinae/Crematogastrini	dulosis	*Tetramoriumhungaricum* Röszler, 1935 Myrmicinae/Crematogastrini
***Strongylognathusitalicus* Finzi, 1924** Vu Myrmicinae/Crematogastrini	dulosis	*Tetramoriumchefketi* Forel, 1911 Myrmicinae/Crematogastrini
***Strongylognathusafer* Emery, 1884** Vu Myrmicinae/Crematogastrini	dulosis	*Tetramoriumhungaricum* Röszler, 1935 Myrmicinae/Crematogastrini
***Bothriomyrmexcommunistus* Santschi, 1919** Dolichoderinae/Bothriomyrmecini	temporary	*Tapinoma* spp. Dolichoderinae/Tapinomini
***Bothriomyrmexcorsicus* Santschi, 1923** Dolichoderinae/Bothriomyrmecini	temporary	*Tapinoma* spp. Dolichoderinae/Tapinomini
***Plagiolepisxene* Stärcke, 1936** Formicinae/Plagiolepidini	inquilinism	*Plagiolepispygmaea* (Latreille, 1798) Formicinae/Plagiolepidini
**Camponotus (Tanaemyrmex) universitatis Forel, 1890** Vu Formicinae/Camponotini	inquilinism	Camponotus (Tanaemyrmex) aethiops (Latreille, 1798) Formicinae/Camponotini
***Lasiuscarniolicus* Mayr, 1861** Formicinae/Lasiini	temporary	formerly *Lasius* s.str. formerly Lasius (Cautolasius) spp. Formicinae/Lasiini
***Lasiusreginae* Faber, 1967** Vu Formicinae/Lasiini	temporary	*Lasiusalienus* (Foerster, 1850) Formicinae/Lasiini
***Lasiusbalcanicus* Seifert, 1988** Formicinae/Lasiini	temporary	formerly *Lasius* s.str. Formicinae/Lasiini
***Lasiusbicornis* (Foerster, 1850)** Formicinae/Lasiini	temporary	formerly *Lasius* s.str. Formicinae/Lasiini
***Lasiuscitrinus* Emery, 1922** Formicinae/Lasiini	temporary	formerly *Lasius* s.str. Formicinae/Lasiini
***Lasiusdistinguendus* (Emery, 1916)** Formicinae/Lasiini	temporary	formerly *Lasius* s.str. Formicinae/Lasiini
***Lasiusmeridionalis* (Bondroit, 1920)** Formicinae/Lasiini	temporary	formerly *Lasius* s.str. Formicinae/Lasiini
***Lasiusmixtus* (Nylander, 1846)** Formicinae/Lasiini	temporary	*Lasiusflavus* (Fabricius, 1782) *Lasiusplatythorax* Seifert, 1991 Formicinae/Lasiini
***Lasiusjensi* Seifert, 1982** Formicinae/Lasiini	temporary	formerly *Lasius* s.str. Formicinae/Lasiini
***Lasiusnitidigaster* Seifert, 1996** Formicinae/Lasiini	temporary	formerly *Lasius* spp. Formicinae/Lasiini
***Lasiussabularum* (Bondroit, 1918)** Formicinae/Lasiini	temporary	formerly *Lasius* spp. Formicinae/Lasiini
***Lasiusumbratus* (Nylander, 1846)** Formicinae/Lasiini	temporary	formerly *Lasius* s.str. Formicinae/Lasiini
***Lasiusfuliginosus* (Latreille, 1798)** Formicinae/Lasiini	temporary	*Lasius* spp. Formicinae/Lasiini
**Formica(s.str.)aquilonia Yarrow, 1955** LR/NT Formicinae/Formicini	temporary	Formica (Serviformica) spp. Formicinae/Formicini
**Formica(s.str.)lugubris Zetterstedt, 1838** LR/NT Formicinae/Formicini	temporary	Formica (Serviformica) spp. Formicinae/Formicini
**Formica(s.str.)polyctena Foerster, 1850** LR/NT Formicinae/Formicini	temporary	Formica (Serviformica) spp. Formicinae/Formicini
**Formica(s.str.)pratensis Retzius, 1783** LR/NT Formicinae/Formicini	temporary	Formica (Serviformica) spp. Formicinae/Formicini
**Formica(s.str.)rufa Linnaeus, 1761** LR/NT Formicinae/Formicini	temporary	Formica (Serviformica) spp. Formicinae/Formicini
**Formica(s.str.)truncorum Fabricius, 1804** Formicinae/Formicini	temporary	Formica (Serviformica) spp. Formicinae/Formicini
**Formica (Coptoformica) exsecta Nylander, 1846** Formicinae/Formicini	temporary	Formica (Serviformica) spp. Formicinae/Formicini
**Formica (Coptoformica) pressilabris Nylander, 1846** Formicinae/Formicini	temporary	Formica (Serviformica) spp. Formicinae/Formicini
**Formica (Raptiformica) sanguinea Latreille, 1798** Formicinae/Formicini	dulosis (facultative)	Formica (Serviformica) spp. Formicinae/Formicini
***Polyergusrufescens* (Latreille, 1798)** Formicinae/Formicini	dulosis	Formica (Serviformica) spp. Formicinae/Formicini
